# Association between serum lipoprotein levels and cognitive impairment in acute cerebral infarction: A protocol for systematic review and meta-analysis

**DOI:** 10.1097/MD.0000000000020178

**Published:** 2020-05-15

**Authors:** Chun-Jie Wei, Chun-Ying Zou, Zeng-Mian Wang, Yao-Jia Jiang

**Affiliations:** a Third Ward of Neurology Department; b Fourth Ward of Neurology Department, First Affiliated Hospital of Jiamusi University, Jiamusi, China.

**Keywords:** acute cerebral infarction, association, cognitive impairment, serum lipoprotein levels

## Abstract

**Background:**

The objective of this study is to examine the association between serum lipoprotein levels (SLL) and cognitive impairment (CI) in patients with acute cerebral infarction (ACI).

**Methods:**

All published studies will be searched from the following electronic databases: PubMed, EMBASE, Cochrane Library, PsycINFO, Web of Science, WANGFANG, and China National Knowledge Infrastructure from inauguration of each electronic database up to March 1, 2020. In addition, we will also search other sources, such as dissertations, Google scholar, conference proceedings, and reference lists of relevant reviews. We will not apply any language restrictions to the electronic databases. Two researchers will independently carry out literature selection, data collection, and methodological quality. A third researcher will help to solve any divergences by discussion. The RevMan 5.3 software will be employed to pool the collected data and to analyze the outcome data.

**Results:**

This study will scrutinize the association between SLL and CI in patients with ACI.

**Conclusions:**

The results of this study will present helpful evidence of the association between SLL and CI in patients with ACI.

Registration number: INPLASY202040018.

## 1 Introduction

Acute cerebral infarction (ACI) is a very common type of stroke, which accounts for about 70% of all stroke patients.^[1,2,3,4]^ It is estimated that ACI may result in 6.2 million deaths around the world each year,^[5,6]^ Patients with such disorder often accompanies a variety of complications, such as paralysis or loss of muscle movement, difficulty talking or swallowing, memory loss or thinking difficulties, pain, emotional problems, and cognitive impairment (CI).^[7,8,9,10,11]^ Of those, CI often greatly affects quality of life in ACI patients.^[12,13]^

Serum lipoprotein has a certain predictive effect on the long-term clinical prognosis of patients with ACI.^[14]^ It pays a very important role in acute cerebral protection and cerebral vascular recovery.^[15]^ Studies have found that CI is associated with serum lipoprotein levels (SLL) in ACI patients.^[16,17,18,19,20,21]^ Previous studies have investigated the association between SLL and CI in patients with ACI. However, no systematic review has been conducted to explore this topic. Therefore, this study will systematically investigate the association between SLL and CI in patients with ACI.

## 2 Methods

### 2.1 Study registration

We have registered this study on INPLASY202040018. It has been organized based on the guidelines of Preferred Reporting Items for Systematic Reviews and Meta-Analysis (PRISMA) Protocol statement.^[22]^

## 3 Criteria for including studies

### 3.1 Types of studies

We will include case-controlled studies that examined the association between SLL and CI in patients with ACI. However, we will exclude animal studies, case report, case series, and uncontrolled clinical studies.

### 3.2 Types of exposures

In the experimental group, all participants experienced ACI.

In the control group, all participants were healthy without ACI.

### 3.3 Types of patients

We will include subjects who had diagnosed as ACI, or who were healthy participants, regardless their race, sex, and age.

### 3.4 Types of outcome measurements

Outcomes are SLL and CI. SLL is examined by enzyme linked immunosorbent assay. CI is measured by any relevant scales, such as Montreal Cognitive Assessment Scale, mini-mental state examination scale, or related tools.

## 4 Data sources and search

The comprehensive search strategy will be carried out in the following electronic databases from their inauguration up to March 1, 2020: PubMed, EMBASE, Cochrane Library, PsycINFO, Web of Science, WANGFANG, and China National Knowledge Infrastructure. We will not apply language and publication time limitations to any literature searches. The sample of search strategy for PubMed is created in Table 1. We will also adapt similar search strategies for other electronic databases.

**Table 1:**
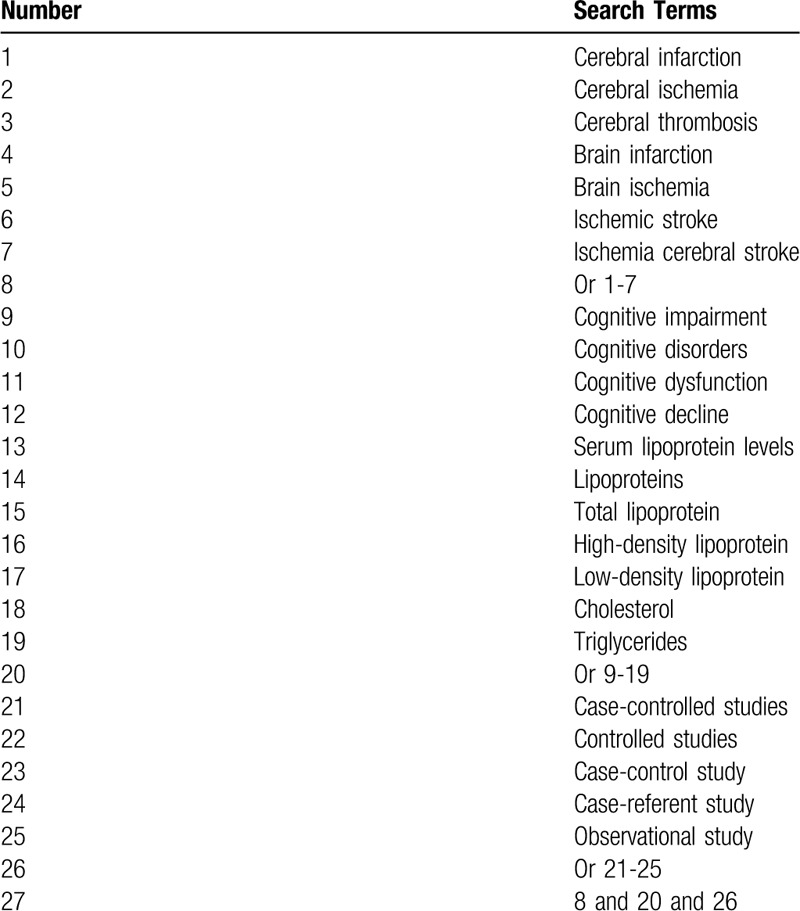
Search strategy for PubMed.

To avoid missing potential studies, we will also check other literature resources, such as Google Scholar, dissertations, conference abstracts, and reference lists of related reviews.

## 5 Data collection and analysis

### 5.1 Study selection

All retrieved citations will be exported into EndNote X7 software and duplicated records will be excluded. Titles and abstracts will be identified by 2 independent investigators, and all unrelated citations will be eliminated. After that, potential articles in full-text will be cautiously examined against all inclusion criteria. Reasons for all exclusions will be recorded and reported. Any disagreements that arise at each stage between two investigators will be solved through consensus with the help of another experienced investigator. We will demonstrate the process of study selection in a PRISMA flow diagram.

### 5.2 Data extraction

Data will be extracted from each eligible study by 2 independent investigators using an advance-designed data extraction sheet. Any differences between two investigators will be resolved by another experienced investigator through discussion. The extracted data includes study information (first author, time of publication, et al), participant characteristics (gender, age, sample size, severity and duration of ACI, et al), study methods, exposures, outcomes, results, findings, and follow-up information. If we identify any missing or insufficient data, we will contact original authors to request it.

### 5.3 Study quality assessment

Two investigators will independently appraise methodological quality for each included study using Newcastle-Ottawa Scale.^[23]^ Any uncertainty between 2 investigators will be solved by another experienced investigator through discussion, and a final decision will be made.

### 5.4 Subgroup analysis

We will conduct subgroup analysis to identify any possible sources of heterogeneity and inconsistency according to the different characteristics of study and patient, and outcomes.

### 5.5 Sensitivity analysis

We will examine sensitivity analysis to test the robustness of conclusions by removing low quality studies.

### 5.6 Reporting bias

We will conduct a funnel plot and Egger regression test to explore any possible reporting bias if over 10 studies are included.^[24,25]^

## 6 Data synthesis

RevMan 5.3 software will be used to pool and to analyze all data in this study. We will express continuous data as weighted mean difference or standardized mean difference and 95% confidence intervals, and dichotomous data as risk ratio and 95% confidence intervals. We will test statistical heterogeneity by *I*^2^ statistics. *I*^2^ ≤ 50% indicates acceptable heterogeneity, and a fixed-effect model will be used, while *I*^2^ > 50% suggests substantial heterogeneity, and a random-effect model will be employed. If *I*^2^ ≤ 50% is identified among sufficient eligible case-controlled studies, we will perform a meta-analysis. On the other hand, if *I*^2^ > 50% is examined, we will carry out a subgroup analysis and sensitivity analysis to explore the sources of heterogeneity.

## 7 Discussion

Several clinical studies have investigated the association between SLL and CI in patients with ACI. So far, no study has been performed to explore the association between SLL and CI in ACI. Therefore, this study will firstly investigate the association between SLL and CI in ACI. This study aims to summarize the most recent evidence of the association between SLL and CI in ACI. The results of this study may provide evidence for clinical practice.

## 8 Ethics and dissemination

This study will not require any ethical approval documents, since it will not collect privacy data. We plan to publish this study on a peer-reviewed journal.

## Author contributions

**Conceptualization:** Chun-Jie Wei, Chun-Ying Zou, Zeng-Mian Wang, Yao-Jia Jiang.

**Data curation:** Zeng-Mian Wang, Yao-Jia Jiang.

**Formal analysis:** Chun-Jie Wei, Chun-Ying Zou, Zeng-Mian Wang.

**Funding acquisition:** Yao-Jia Jiang.

**Investigation:** Yao-Jia Jiang.

**Methodology:** Chun-Jie Wei, Chun-Ying Zou, Zeng-Mian Wang.

**Project administration:** Yao-Jia Jiang.

**Resources:** Chun-Ying Zou, Zeng-Mian Wang.

**Software:** Chun-Jie Wei, Chun-Ying Zou, Zeng-Mian Wang.

**Supervision:** Yao-Jia Jiang.

**Validation:** Chun-Jie Wei, Chun-Ying Zou, Zeng-Mian Wang, Yao-Jia Jiang.

**Visualization:** Chun-Jie Wei, Chun-Ying Zou, Yao-Jia Jiang.

**Writing – Original Draft:** Chun-Jie Wei, Yao-Jia Jiang.

**Writing – Review & Editing:** Chun-Jie Wei, Chun-Ying Zou, Zeng-Mian Wang, Yao-Jia Jiang.

## References

[1] Sun Z Xu Q Gao G Clinical observation in edaravone treatment for acute cerebral infarction. Niger J Clin Pract 2019;22:1324–7. 31607719 10.4103/njcp.njcp_367_18

[2] Lyu DP Wang Y Wang K Acute cerebral infarction in a patient with persistent trigeminal artery and homolateral hypoplasia of internal carotid artery distal anastomosis: a case report and a mini review of the literature. J Stroke Cerebrovasc Dis 2019;28:104388. 31575472 10.1016/j.jstrokecerebrovasdis.2019.104388

[3] Qin C Shang K Xu SB Efficacy and safety of direct aspiration versus stent-retriever for recanalization in acute cerebral infarction: A PRISMA-compliant systematic review and meta-analysis. Medicine (Baltimore) 2018;97:e12770. 30313091 10.1097/MD.0000000000012770 PMC6203566

[4] Cheng J Zhou ZW Sheng HP An evidence-based update on the pharmacological activities and possible molecular targets of Lycium barbarum polysaccharides. Drug Des Devel Ther 2015;9:33–78. 10.2147/DDDT.S72892 PMC4277126 25552899

[5] Inoue T Kobayashi M Uetsuka Y Pharmacoeconomic analysis of cilostazol for the secondary prevention of cerebral infarction. Circ J 2006;70:453–8. 16565564 10.1253/circj.70.453

[6] You YN Cho MR Kim JH Assessing the quality of reports about randomized controlled trials of scalp acupuncture combined with another treatment for stroke. BMC Complement Altern Med 2017;17:452. 28877716 10.1186/s12906-017-1950-6 PMC5588620

[7] Zeng Q Huang Z Wei L Correlations of serum cystatin C level and gene polymorphism with vascular cognitive impairment after acute cerebral infarction. Neurol Sci 2019;40:1049–54. 30805744 10.1007/s10072-019-03777-8

[8] Chu H Zhang S Fu J TIE's flying acupuncture for acute cerebral infarction hemiplegia: a randomized controlled trial. Zhongguo Zhen Jiu 2017;37:1153–6. 29354949 10.13703/j.0255-2930.2017.11.004

[9] Chen X Bi H Zhang M Research of sleep disorders in patients with acute cerebral infarction. J Stroke Cerebrovasc Dis 2015;24:2508–13. 26316356 10.1016/j.jstrokecerebrovasdis.2015.06.033

[10] Saito T Hayashi K Nakazawa H Clinical characteristics and lesions responsible for swallowing hesitation after acute cerebral infarction. Dysphagia 2016;31:567–73. 27277890 10.1007/s00455-016-9716-8 PMC4938849

[11] Houston JG Morris AD Grosset DG Ultrasonic evaluation of movement of the diaphragm after acute cerebral infarction. J Neurol Neurosurg Psychiatry 1995;58:738–41. 7608679 10.1136/jnnp.58.6.738 PMC1073558

[12] Zhen X Zheng Y Hong X Physiological ischemic training promotes brain collateral formation and improves functions in patients with acute cerebral infarction. Front Neurol 2016;7:235. 28066319 10.3389/fneur.2016.00235 PMC5177612

[13] Zhang W Huang Y Li Y Efficacy and safety of vinpocetine as part of treatment for acute cerebral infarction: a randomized, open-label, controlled, multicenter CAVIN (Chinese assessment for vinpocetine in neurology) trial. Clin Drug Investig 2016;36:697–704. 10.1007/s40261-016-0415-x 27283947

[14] Guo AS Li AH Chen X Effect of acupoint catgut embedding on motor function and serum high sensitivity C-reactive protein and IL-6 levels in patients with acute cerebral infarction. Zhen Ci Yan Jiu 2013;38:224–8. 24006669

[15] Li L Ren S Hao X Efficacy of minimally invasive intervention in patients with acute cerebral infarction. J Cardiovasc Pharmacol 2019;73:22–6. 30540689 10.1097/FJC.0000000000000625

[16] Ren HY To explore the relationship between serum lipid levels and NIHSS scores in patients with acute cerebral infarction. Modern Chin Med Appl 2017;11:31–2.

[17] Ren HY Jia JR Liu Z Correlation between serum lipoprotein levels and cognitive impairment in patients with acute cerebral infarction. China Cont Med Educ 2016;8:59–60.

[18] Wang YX Correlation of serum lipoproteins, high-sensitivity C-reactive protein and thrombus precursor protein with carotid atherosclerotic plaque in patients with acute cerebral infarction. Chin J Gerontol 2016;36:2908–9.

[19] Li XP Changes in serum lipoprotein levels in patients with acute cerebral infarction and their effects on carotid atherosclerotic plaque formation and stability. Chin J Pract Nerv Dis 2016;19:103–4.

[20] Ren HY Lin LM Study on serum levels of lipoproteins and their influencing factors in patients with acute cerebral infarction. J Baotou Med Coll 2011;27:24–6.

[21] Qi Y Li DY Serum levels of lipoproteins and their correlation in patients with acute cerebral infarction. J Lanzhou Univ (Med Ed) 2010;36:54–7.

[22] Shamseer L Moher D Clarke M PRISMA-P Group. Preferred reporting items for systematic review and meta-analysis protocols (PRISMA-P) 2015: elaboration and explanation. BMJ 2015;349:g7647. 10.1136/bmj.g7647 25555855

[23] Stang A Critical evaluation of the Newcastle-Ottawa scale for the assessment of the quality of nonrandomized studies in meta-analyses. Eur J Epidemiol 2010;25:603–5. 20652370 10.1007/s10654-010-9491-z

[24] Sutton AJ Duval SJ Tweedie RL Empirical assessment of effect of publication bias on meta-analyses. BMJ 2000;320:1574–7. 10845965 10.1136/bmj.320.7249.1574 PMC27401

[25] Egger M Davey Smith G Schneider M Bias in meta-analysis detected by a simple, graphical test. BMJ 1997;315:629–34. 9310563 10.1136/bmj.315.7109.629 PMC2127453

